# Schizophrenia Assessment, Referral and Awareness Training for Health Auxiliaries (SARATHA): Protocol for a Mixed-Methods Pilot Study in Rural India

**DOI:** 10.3390/ijerph192214936

**Published:** 2022-11-13

**Authors:** John A. Naslund, Vidhi Tyagi, Azaz Khan, Saher Siddiqui, Minal Kakra Abhilashi, Pooja Dhurve, Urvakhsh Meherwan Mehta, Abhijit Rozatkar, Urvita Bhatia, Anil Vartak, John Torous, Deepak Tugnawat, Anant Bhan

**Affiliations:** 1 Department of Global Health and Social Medicine, Harvard Medical School, Boston, MA 02115, USA; 2 Sangath, Bhopal 462042, India; 3 Icahn School of Medicine at Mount Sinai, New York, NY 10029, USA; 4 Sangath, New Delhi 110030, India; 5 Department of Psychiatry, National Institute of Mental Health and Neurosciences (NIMHANS), Bengaluru 560029, India; 6 Department of Psychiatry, All India Institute of Medical Sciences (AIIMS), Bhopal 462026, India; 7 Department of Psychology, Health and Professional Development, Oxford Brookes University, Oxford OX3 0BP, UK; 8 Sangath, Porvorim 403501, India; 9 Schizophrenia Awareness Association, Pune 411041, India; 10 Beth Israel Deaconess Medical Center, Boston, MA 02215, USA

**Keywords:** community health workers, schizophrenia, task-sharing, severe mental disorders, digital technology

## Abstract

Background: Workforce shortages pose major obstacles to the timely detection and treatment of schizophrenia, particularly in low-income and middle-income countries. The SARATHA (Schizophrenia Assessment, Referral, and Awareness Training for Health Auxiliaries) project involves the systematic development, iterative refinement, and pilot testing of a digital program for training community health workers in the early detection and referral of schizophrenia in primary care settings in rural India. Methods: SARATHA is a three-phase study. Phase 1 involves consulting with experts and clinicians, and drawing from existing evidence to inform the development of a curriculum for training community health workers. Phase 2 consists of designing and digitizing the training content for delivery on a smartphone app. Design workshops and focus group discussions will be conducted to seek input from community health workers and service users living with schizophrenia to guide revisions and refinements to the program content. Lastly, Phase 3 entails piloting the training program with a target sample of 20 community health workers to assess feasibility and acceptability. Preliminary effectiveness will be explored, as measured by community health workers’ changes in knowledge about schizophrenia and the program content after completing the training. Discussion: If successful, this digital training program will offer a potentially scalable approach for building capacity of frontline community health workers towards reducing delays in early detection of schizophrenia in primary care settings in rural India. This study can inform efforts to improve treatment outcomes for persons living with schizophrenia in low-resource settings.

## 1. Introduction

Globally, workforce shortages pose major obstacles to the timely detection and treatment of severe mental disorders, including schizophrenia and other psychoses [1,2,3]. In most countries, schizophrenia is among the leading contributors to years lived with disability due to mental disorders [3]. Individuals living with schizophrenia experience deficits in functioning, persistent and debilitating symptoms, and premature mortality when compared to the general population [4,5]. These individuals also face societal stigma and discrimination [6], making it difficult to seek care. In low-income and middle-income countries (LMICs), the gap in available mental health services is greatest, where the vast majority of individuals living with schizophrenia do not have access to basic and essential care [1]. Significant delays in the early detection of schizophrenia results in further worsening of symptoms and deterioration in functioning with detrimental consequences for individuals, their families, and society, highlighting the urgency to ensure timely delivery of evidence-based care to this patient group [7].

Task sharing, which involves training and building capacity of community health workers, holds potential to tackle these significant workforce shortages [8]. For instance, in resource-poor settings, task sharing has emerged as an effective approach to addressing health workforce challenges and supporting the delivery of mental health services in primary care [9,10]. Task sharing includes shifting service delivery of specific tasks from professionals with specialized training to those without any prior training or experience in mental health care, such as community health workers, by facilitating training, supervision, and ongoing support for this new cadre of mental health providers [8]. To ensure that patient outcomes are not compromised in this endeavor, as an important first step it is vital to appropriately train community health workers in the delivery of mental health care, particularly for supporting care for this vulnerable patient population of persons living with schizophrenia. Such community-based programs, focusing on reducing disability through community mobilization and individual and family support, are recommended by the World Health Organization (WHO) as psychosocial intervention for delivery alongside facility-based care and antipsychotic medication for people with schizophrenia [11,12].

In recent years, there have been various community models of care delivery involving task-sharing that have shown promise for treating severe mental disorders such as schizophrenia in primary care settings in LMICs [13]. For instance, the “Community care for People with Schizophrenia in India (COPSI)” is a task-sharing model involving a collaborative community-based care intervention combined with standard facility care [14,15]. Specifically, the program was delivered by trained community health workers, and emphasized collaboration between the individual with schizophrenia, their caregivers, and the treatment team [14]. In a randomized trial, the COPSI program achieved improvements in the positive and negative syndrome scale (PANSS) and the Indian disability evaluation and assessment scale (IDEAS), and the program was found to be comparable to a standard facility care control condition in terms of the proportion of participants who experienced a reduction of more than 20% in overall symptoms [14]. Nevertheless, while the community intervention with standard facility care was modestly more effective than facility care alone, these results highlight the promise of community-based interventions, particularly towards improving access to care for this at-risk patient population in low-resource settings in India.

In the “Rehabilitation Intervention for people with Schizophrenia in Ethiopia (RISE)” study, a community-based rehabilitation intervention was evaluated in combination with facility-based care consisting of a task-shared model of mental health care integrated within primary care in rural districts [16]. The community-based rehabilitation program focuses on reducing disability through community mobilization and individual and family support, and is recommended by the WHO as a psychosocial intervention for delivery alongside facility-based care and antipsychotic medication for people with schizophrenia [11,12]. Specifically, in the RISE study, the community-based rehabilitation intervention was led by lay workers and involved home visits with a focus on psychoeducation, family intervention, crisis management, and support for returning to work, in addition to community mobilization. The program had a positive impact on functioning, yet factors such as poverty and lack of access to antipsychotic medications remained challenges. The acceptability of the community-based rehabilitation intervention was first demonstrated in a mixed methods pilot study [16], and a subsequent randomized controlled trial demonstrated the effectiveness of the community-based rehabilitation program combined with facility-based care in significantly reducing disability as measured by the WHO Disability Assessment Schedule (WHODAS) 2.0 among persons with schizophrenia when compared to facility-based care alone [17]. The RISE study offered further compelling evidence on the acceptability and effectiveness of a community-based rehabilitation intervention delivered by lay health workers in combination with facility-based care for schizophrenia [17], and highlights important opportunities to build capacity of frontline health workers for early identification and referral as well as supporting rehabilitation and recovery for this at-risk patient population.

With increasing access to and use of digital technologies globally, there are opportunities to leverage these technologies for supporting frontline health workers as part of task sharing efforts, and in particular for supporting community-based care for schizophrenia [8,18,19]. In a study conducted in rural China, digital technology was used as part of the “Lay Health Supporters, E-platform, Award, and iNtegration” (LEAN) project to support lay providers, consisting of family members and community volunteers, in caring for persons living with schizophrenia in the community [20]. The LEAN digital platform uses mobile text messaging to assist lay providers with supervising medication use, providing reminders and health education, monitoring for signs of relapse, and enabling connection with local village doctors and referral to specialty psychiatric care when needed. When compared to a usual care control condition, the LEAN intervention group demonstrated higher rates of medication adherence, reduction in risk of relapse, and lower re-hospitalization rates.

These studies underscore various aspects of task-sharing that are pertinent in the ongoing treatment of individuals living with schizophrenia in LMICs, yet further consideration is needed regarding ways to expand the reach and potential impact of these community-based interventions earlier in the course of the illness. For instance, it will be important to consider how technology can support the early detection of schizophrenia, given that delays in initiating treatment represent a major contributor to poorer outcomes and greater disability [21]. Given recent promising examples of leveraging digital technologies for supporting care for severe mental disorders, including schizophrenia, in LMICs [18,22,23], there may be new opportunities to build on these existing studies to capitalize on the increasing availability of digital devices to advance task sharing efforts by facilitating the training of community health workers [24]. Few studies have specifically focused on the use of widely available digital technologies for developing the skills and competencies of community health workers for supporting care for schizophrenia [18]. Therefore, this exploratory mixed methods study, called SARATHA (“Schizophrenia Assessment, Referral, and Awareness Training for Health Auxiliaries”) seeks to enable the systematic development, iterative refinement, and pilot testing of a digital program for training community health workers in the early detection and referral of cases of schizophrenia in primary care settings in rural India.

### Study Objectives

The SARATHA project consists of the following three objectives: (1) to develop the curriculum for the digital program for training community health workers in the early detection and referral, and support treatment of persons with schizophrenia in primary care settings in rural India. This will require adapting content from existing evidence-based community programs for treatment of schizophrenia, and collecting feedback and recommendations from experts and clinicians; (2) to develop the digital training content following a step-wise approach. This process involves writing scripts for the content, obtaining expert feedback, and working with community health workers to refine the content and ensure acceptability for the target setting and context. As part of this second objective, service users living with schizophrenia will also be engaged in reviewing the content to collect their feedback and insights to further ensure relevance of the program for supporting the target patient population; and (3) to test the feasibility and acceptability of the digital training program with a sample of 20 community health workers. Community health workers will participate in qualitative focus group discussions to collect their perspectives about the feasibility and acceptability of the digital training, and to elicit feedback for improving the program design and content and supporting program integration into their clinic workflow. Preliminary effectiveness of the training program will be explored, as measured using assessments of participants’ knowledge and skills, and general attitudes about schizophrenia before and after completing the training program.

## 2. Methods

### 2.1. Setting

The SARATHA project will be conducted in the Sehore district of Madhya Pradesh, India. Madhya Pradesh is one of the largest states of India, with a population over 72 million, nearly 73% of whom live in rural areas [25]. This state lags behind many other Indian states and was ranked 20 (out of 23 states) on the Human Development Index [26] and last on the Global Hunger Index compared to other Indian states [27]. Recent reports have shown that the care gap for mental health problems in Madhya Pradesh exceeds 91% [2], and per capita disability attributed to mental disorders is greater in Madhya Pradesh relative to most other states [3].

This study is led by Sangath, in collaboration with researchers at Harvard Medical School in the United States. Sangath is a leading non-profit research organization in India that has established research infrastructure in the region of Madhya Pradesh, and collaborations with the All India Institute of Medical Sciences (AIIMS) Bhopal, and the National Institute of Mental Health and Neurosciences, apart from other hubs elsewhere in the country. Sangath has a longstanding collaboration with the Department of Public Health and Family Welfare, Government of Madhya Pradesh, beginning in 2011 with the roll out of the India site of PRIME (Program for Improving Mental health Care), an eight-year research consortium funded by UKAid [28,29]. This collaboration with the Department of Public Health and Family Welfare led, in 2015, to the launching of the Scaling up Opportunities for Healthy and Active Minds (SOHAM) initiative [29], with the goal of extending mental health care throughout the state through the Government’s district health care system. Sangath is now leading the National Institute of Mental Health (NIMH) funded ESSENCE (Enabling translation of Science to Service to Enhance Depression Care) project, supported by a Memorandum of Understanding with the National Health Systems Resource Centre (NHSRC), Government of India and Department of Public Health and Family Welfare, Government of Madhya Pradesh, a major component of which seeks to leverage digital technology for building capacity of community health workers in treating common mental disorders in primary care settings [30]. More recently, Sangath has been collaborating with Beth Israel Deaconess Medical Center and Harvard Medical School in the United States, and AIIMS Bhopal and NIMHANS in India to support an effort aimed at leveraging smartphone technology for digital phenotyping in patients with schizophrenia as part of the SHARP (Smartphone Health Assessment for Relapse Prevention) project [31,32,33,34,35]. The SARATHA project builds on these prior and ongoing efforts, and involves recruiting community health workers from primary healthcare facilities in the Sehore district who are already enrolled in other ongoing training activities [30].

### 2.2. Participants for the Training Program

The target health workers for the training program being developed in the SARATHA project include community health workers, referred to as ASHAs—Accredited Social Health Activists, and ASHA Facilitators. ASHAs and ASHA Facilitators are all women; they work at the primary care or community levels, and do not currently receive specialized training in delivering mental health care, or in the detection and referral of severe mental disorders such as schizophrenia. ASHAs are deployed within India’s National Health Mission, and are selected from the community they serve primarily to promote facility-based childbirth [36]. Each ASHA covers a population of about 1000 and receives a minimum monthly remuneration as well as performance-based and service-based compensation for facilitating immunization, and referral and escort services for facility-based childbirth. Each ASHA also receives 23 days of training in the first year and 12 days of training every subsequent year thereafter. The ASHA Facilitator is responsible for monitoring, providing supportive supervision and on-site assistance for the ASHAs. One ASHA Facilitator is expected to support approximately 15–20 ASHAs. The ASHA Facilitator serves as the link between the ASHA and the facilities for supporting delivery of care at the community-level. For this study, we will recruit ASHAs and ASHA Facilitators currently enrolled in an ongoing research project evaluating a digital program for training in the delivery of depression care [30]; therefore, they will have had some prior exposure to methods for treating common mental disorders such as depression before learning the content focused on schizophrenia in the current study.

### 2.3. Study Design

**Figure 1:**
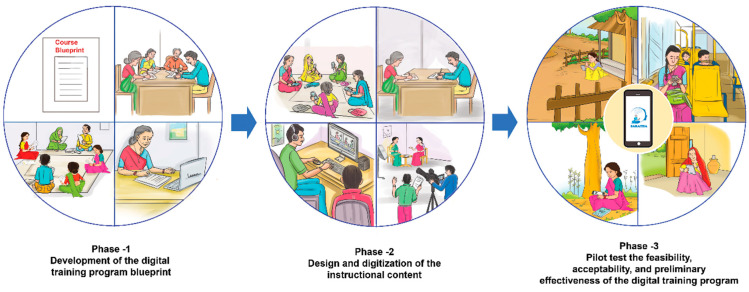
Stepwise approach for intervention development.

The SARATHA study employs a mixed-methods approach to ensure that feedback collected from experts, community health workers, and service users living with schizophrenia can be incorporated into the design, development and evaluation of this training program. This study leverages increasingly available smartphone technology to develop capacity and support the acquisition of skills among community health workers for the detection, creating awareness, providing mental health first aid, and facilitating referral of patients with schizophrenia in primary care settings in rural India. This study consists of three parts aligned with the project aims, as illustrated in Figure 1, that will occur sequentially as follows:

#### 2.3.1. Phase 1—Development of the Digital Training Program Curriculum

The first part of the study involves engaging subject matter experts and clinicians from within the network of collaborators of Harvard Medical School and Sangath consisting of clinicians and researchers with expertise in the detection, diagnosis, and treatment of severe mental disorders, including schizophrenia, in low-resource settings. These experts will review and provide guidance on the design of the curriculum that will serve as the blueprint for designing the digital training program for community health workers. Through multiple rounds of feedback followed by revisions, the course curriculum will be finalized.

#### 2.3.2. Phase 2—Design and Digitization of the Instructional Content

This second part of the study involves development and digitizing of the training program content and collecting feedback from community health workers to refine and make improvements to the content. Following a step-wise approach that our team has previously applied to the development of a digital program for training community health workers in the delivery of a brief psychological treatment for depression [37], we will draw from the person-based approach to intervention development [38] as well as being informed by the ADDIE (Analysis, Design, Develop, Implement, and Evaluation) framework [39]. The ADDIE framework is a recognized model for guiding instructional design [39,40], and is relevant for supporting the development of digital training programs because it allows flexibility and multiple iterations in the program content and design to reach a final program for pilot testing. This will guide our development of the scripts for the training program content, adhering to the blueprint developed in the first aim of the study [37], while simultaneously incorporating user feedback to ensure usability and relevance for our target group of community health workers. The scripts will be reviewed by content experts to ensure fidelity to the evidence-based program content. For this program, the content will be adapted from the COPSI manuals [41] and supplemented with content from the Department of Empowerment of Persons with Disability [42], the PREMIUM Counselling Relationship Manual [43], and the National Institute of Mental Health and Neurosciences [44], and will be translated into Hindi. Community health workers will participate in the review and translation of the scripts to ensure that the use of language and terms is contextually and culturally relevant for the target setting.

Next, film production and digitizing the content will begin. To support this process, we will organize a series of design workshops followed by qualitative focus group discussions to elicit deeper insights from community health workers (not involved in the script review process). Specifically, small groups of approximately 8–10 community health workers (ASHAs or ASHA facilitators) will be invited to comment on the digital lesson content, offering feedback and recommendations for ensuring that the content is interesting and engaging, relatable, of relevance, and acceptable for the target audience. Additionally, a group of service users, i.e., individuals who self-identify as living with schizophrenia, will be invited to comment on the content of the digital training program to ensure that it is relevant and appropriate for supporting for this target patient population. Throughout this process, COVID-19 safety measures, as determined by local health authority, will be followed during any in-person interactions [45].

During these design workshops, the research team will present the digital content to the community health workers. The digital content will consist of short videos (lectures and role-plays), PowerPoint presentations, images and graphics, and brief learning activities, to obtain their feedback and recommendations. By directly involving community health workers in designing and developing the digital training program, the goal is to maximize uptake of the training program and to ensure program relevance for reaching out to persons who may have schizophrenia in community settings and responding to the concerns of their caregivers. This approach expands on recent successful efforts by our team to involve community health workers in the development of digital training for depression treatment as part of the ESSENCE project [30,37,46,47]. Furthermore, this systematic approach for designing the digital content will help to ensure that the training program is culturally and contextually relevant, considering the views and experiences of community health workers, making sure the language is clear and approachable, while closely following the curriculum informed by the experts and clinicians in the first phase. The final digital training content will be uploaded to the Sangath Learning Management System, a digital education platform built on the Moodle software platform with core features such as creating course structure, hosting digital content, designing interactive activities, working both online and offline, ability to track learner progress, and accessible by a smartphone application in preparation for pilot testing [37].

#### 2.3.3. Phase 3—Pilot Test the Feasibility, Acceptability, and Preliminary Effectiveness of the Digital Training Program

This third part of the project focuses on evaluating feasibility, acceptability, and preliminary effectiveness of the digital training program with 20 community health workers. These community health workers will be separate from those who previously contributed to the digital program development (in the second phase). To facilitate recruitment, community health workers who have completed training in delivery of depression care as part of the ESSENCE project will be invited to participate in this pilot study [30]. These community health workers will be invited to complete the digital training on detection and referral of schizophrenia in primary care settings developed in Phase 2. The training will include a brief in-person orientation to the digital technology, purpose of the training program, and tips for navigating the smartphone and features of the app for accessing the learning content. These community health workers will have up to 4 weeks to complete the digital training, and will complete a brief pre-post knowledge assessment to assess initial program impact reflected as change in their: (1) general knowledge about schizophrenia, and associated symptoms; (2) approaches for detection and referral of schizophrenia in primary care and community settings; and (3) methods for communicating with and offering support to family caregivers of persons with schizophrenia. The assessment is self-report, consisting of multiple-choice questions following a similar approach that our team has employed for knowledge assessment among community health workers [48]. The community health workers will also complete questionnaires to rate the usability and acceptability of different aspects of the instructional content of the program and layout of the digital app, and will be invited to participate in exit focus group discussions at the conclusion of the training to further assess program feasibility and acceptability by exploring their perceptions and understanding about schizophrenia, their general feedback about the course design, content and duration, and their views about how to integrate this training and care for schizophrenia into their work in community settings.

### 2.4. Evidence-Based Content to Adapt for the Training Program

The program content utilized in SARATHA is primarily adapted from the COPSI manual, a guide for working with patients with schizophrenia and their families in low-resource settings in India [14]. The aim of the COPSI program guide is for community health workers to use this in combination with formal medical treatment provided by specialist providers such as psychiatrists. This can support community health workers in identifying and providing basic mental health first aid to individuals living with schizophrenia in their communities, while actively involving caregivers in the treatment process. The COPSI manual is an extensive guide that is broken down into different modules. The COPSI program introduces schizophrenia and tackles topics including symptoms, the course and outcome of the disease, disabilities caused by the disease, and impact on caregivers. Furthermore, the COPSI program covers principles and methods of providing care for patients with schizophrenia, and provides an overview of individualized treatments, principles of counseling, family support, stigma and discrimination, and available medical treatment. This also includes content pertaining to topics like assessment and management of suicide risk, delivery of family intervention, adherence management, and rehabilitation, as well as various resources such as initial contact and follow-up forms, and checklists for documenting medication side effects and assessing suicide risk.

As part of the SARATHA project, we have combined the core content of the COPSI manual, and elaborated on specific sections relevant for the role of the target group of community health workers. This content was also supplemented with content from the Department of Empowerment of Persons with Disability [42], PREMIUM Counselling Relationship Manual [43], and the National Institute of Mental Health and Neurosciences [44]. This has resulted in the development of a brief 6-module program, with the curriculum and content summarized in Table 1. The program covers several key topics, beginning with a brief overview of schizophrenia and its symptoms, and the phases of illness, as well as common misconceptions about persons living with schizophrenia and issues pertaining to stigma and discrimination. The various types of available medical and psychosocial treatments are described, along with common physical comorbidities with schizophrenia, and consideration of rehabilitation programs and potential disability schemes and benefits. Given that community health workers have played a critical role in the COVID-19 response in India, there is also content describing the impact of COVID-19 on patients with schizophrenia as they represent a particularly vulnerable patient population. The program also emphasizes specific skills, such as screening and ways to identify potential schizophrenia cases in the community, managing suicide risk, and how to respond to family members and caregivers by delivering mental health first aid. Another key component of the training relates to how to make a referral, which involves coordinating with clinicians and caregivers, documentation and reporting, and post referral follow-ups.

### 2.5. Ethical Approval

Ethical approval for this study was obtained from the Institutional Review Boards at Sangath, India (Number: JN_2019_64) and Harvard Medical School, USA (Number: IRB20-1164).

## 3. Discussion

The early identification of schizophrenia in the community and initiation of treatment represents a global public health priority. This is an urgent challenge in LMICs, where extended delays in detecting and treating schizophrenia are associated with detrimental outcomes for individuals, as well as negative consequences to their families, communities, and society at large [7]. This project represents an initial step towards addressing this gap by building the capacity of community health workers in rural India, who may be ideally positioned to support the screening and referral of potential cases to the limited number of available mental health specialists, while offering brief psychosocial support to family members of persons with a probable diagnosis of schizophrenia experiencing distress, as well as serving as a link between the community and the health system. The SARATHA project described in this protocol is novel because it seeks to leverage widely available digital technology to facilitate the training of community health workers including ASHAs and ASHA facilitators, an essential frontline provider workforce within India’s National Health Mission [36,49], to act as the primary touch point for persons living with schizophrenia within the community. This study holds potential to break new ground towards reducing the time lag in identifying and initiating treatment for individuals living with schizophrenia, including those experiencing first episode psychosis, while connecting these individuals with available services and offering support to their families.

This study could also serve as a critical juncture for accelerating access to rehabilitation services, and other potentially available community supports over time, thereby enabling individuals living with schizophrenia to engage in community activities and empowering family members in managing their care towards improving overall functioning. There may be opportunities to expand on the work described in this protocol to support community rehabilitation following hospitalization. For instance, a study from China evaluated the effectiveness of a family intervention, involving both individual and group counseling emphasizing education about schizophrenia and strategies for responding to and supporting the patient within the community following hospitalization [50]. This study from China demonstrated that with adequate support, family members can be made aware of how to offer care and support to their loved one who was hospitalized for schizophrenia, whereby patients who received the family support intervention displayed significantly lower rates of re-hospitalization and demonstrated considerably higher overall functioning when compared to a usual care control condition [50]. While our study involves training community health workers to offer basic support to family members of individuals living with schizophrenia, there may be opportunities to expand on this connection to further the reach of community-based rehabilitation services and natural support for persons living with schizophrenia. In another example from Iran, the feasibility and effectiveness of aftercare services following initial treatment for schizophrenia was demonstrated in community-based settings [51]. In this study from Iran, the provision of aftercare services resulted in a reduction in the number of rehospitalizations and reduced length of hospital stay among adult patients living with schizophrenia and other severe mental disorders [51]. Similarly, these successful aftercare services included treatment follow-up, family psychoeducation, and social skills training.

In the context of India, the Schizophrenia Research Foundation (SCARF) is a non-governmental organization engaged in supporting access to care for persons living with schizophrenia as well as research pertaining to schizophrenia spectrum disorders [52]. SCARF has been spearheading the Family Education Program initiative for over 10 years, which seeks to evaluate the efficacy of structured psycho-education for families of patients living with chronic schizophrenia [53]. The program consists of six sessions delivered over a 6-week period to primary caregivers of patients living with schizophrenia [53]. In a preliminary evaluation, the information imparted during the sessions was well received, and the sessions provided a forum for information sharing and interaction between families where issues pertaining to schizophrenia were raised and discussed. This contributed to an increase in knowledge about schizophrenia as evidenced during feedback interviews with primary caregivers, suggesting the program may have been valuable in terms of knowledge gains and practical applications [53].

In another example from India, patients with schizophrenia and their caregivers were randomly allocated to receive either a structured psychoeducational intervention consisting of monthly sessions or ‘routine’ outpatient care for 9 months; and carried out by two mental health professionals and were trained for two months by psychiatrists, and psychopathology was assessed monthly [54]. Disability levels, caregiver-burden, caregiver-coping, caregiver-support, and caregiver-satisfaction were evaluated at baseline and upon program completion. The structured intervention had two phases; first, an engagement phase to build positive therapeutic alliance with family and provide preliminary information about schizophrenia with 1–2 sessions each month; and then, an active intervention phase lasting 9 months with monthly sessions of 40–60 min with caregivers including education about etiology, symptoms, treatment and prognosis, medication management, realistic goal setting, communication and problem solving training, identification of early signs of relapse, disability benefits, and accessibility to mental health facilities. The structured psychoeducational intervention was significantly better than routine outpatient care on several indices including psychopathology, disability, caregiver support and caregiver satisfaction [54]. Another study similarly assessed the efficacy of family psychoeducation interventions on the caregivers of patients with schizophrenia with respect to their perceived quality of life [55]. A total of 30 caregivers of male patients living with schizophrenia were selected and divided into either a family psychoeducation intervention given on a bi-monthly basis for 6 months, or control condition [55]. Following family psychoeducation, significant improvement in overall quality of life scores was observed in the experimental group caregivers compared to the control group caregivers where no such intervention was provided.

While the current focus of SARATHA is to train community health workers to detect and refer patients with possible signs of schizophrenia across rural settings, similar to the studies discussed above, it also contains modules that emphasize psychoeducation and support to manage distress among family members and caregivers. Furthermore, a future direction for SARATHA may be the addition of more extensive content emphasizing caregiver education, with specific outcome measures, such as changes in family attitudes, to measure its success. Our study also expands on other ongoing efforts in India aimed at building capacity among community health workers including ASHAs to support the community-based rehabilitation for persons with severe mental disorders. An ongoing study in Karnataka is evaluating whether a community-based rehabilitation program delivered by ASHAs in addition to normal treatment is more effective in reducing disabilities caused by severe mental disorders relative to usual treatment [56]. The secondary outcomes measured will include duration of clinical remission, work status and income, self-stigma, and community attitudes toward the person living with the illness. This study will shed light on methods of delivering community-based rehabilitation for persons living with severe mental disorders through ASHAs with potential universal applicability.

The various types of extended services illustrated in these various examples are beyond the initial scope of the SARATHA study; yet, these studies offer valuable evidence to suggest that community providers and lay providers in LMICs may be ideally positioned to offer ongoing support to individuals living with schizophrenia and their family members to improve functioning, community tenure, and prevent rehospitalization. SARATHA similarly focuses on building the capacity of community health workers, taking this initiative a step further by leveraging widely available smartphone technologies, which hold potential for wide scalability, to bridge gaps in care among vulnerable patient communities suffering from severe mental disorders. The findings from this pilot study will add to mounting evidence highlighting the key role for the inclusion of mobile technologies in the training of community health workers [24,57].

Another key consideration is the potential impact of the SARATHA project on stigma and discrimination within the community, and overall perceptions and response to persons living with schizophrenia, or suspected as living with schizophrenia, and their family members. The stigma and discrimination associated with mental illness are often devastating and can be detrimental to recovery [58]. Discrimination is the action based on stigma that can be directed toward the stigmatized [59] and reflects how a person acts after discovering that someone has a mental illness. Around the world, people living with schizophrenia face a disproportionate burden of stigma and discrimination, have restricted work opportunities, and are even denied many basic rights afforded to other members of society [6,59]. The stigma and discrimination associated with schizophrenia are seen to have a prominent effect on the lives of those suffering from the illness, and are driven by a lack of knowledge, and symptoms associated with the illness [6]. By educating frontline providers about the different symptoms, as well as the treatment options, and the possibility to achieve recovery through support and access to community rehabilitation, it may be possible to further address the detrimental consequences of mental health stigma. Follow up studies will be necessary to determine if building capacity of frontline community health workers can mitigate the harmful effects of stigma and misconceptions about schizophrenia within the communities they serve.

### Limitations

There are limitations with the SARATHA study that warrant consideration. First, this represents a preliminary study aimed at developing and pilot testing a training program for community health workers in rural India. Therefore, the study is not sufficiently powered to detect an effect and to determine the effectiveness of the digital training program. Second, the outcomes in this study are focused on knowledge about schizophrenia and satisfaction with the training program and will be collected through self-report. These measures will not offer objective assessment of health worker performance delivering the content covered in the training program within community settings, which limits the external validity of this study. This also highlights an important area for future investigation to expand on this preliminary work by evaluating whether community health workers can effectively apply the skills gained through this training program within their regular clinical work. Third, there may be limits to the generalizability of the findings from this study. While this study will be conducted in one rural district in a large state in Central India, which may be representative of a large segment of the population in India, it is not possible to determine whether these findings will be similarly relevant across other settings in India or globally. To address this concern, our team is working with other organizations across India as well as internationally to comment on the training program materials and study design and offer recommendations for how these findings can be applied to diverse settings.

Furthermore, the community health workers who will enroll in this study will already have had prior exposure to mental health training content focused on depression. As a result, the target sample of community health workers may be more open to learning about severe mental disorders such as schizophrenia. Fourth, in this study we specifically targeted ASHAs given that they represent the largest workforce of community health workers in India, numbering close to 1 million and deployed across most states in the country [36,49]. However, this limits whether the findings may generalize to other cadres of health workers or lay providers, who could also be ideally positioned to learn and apply the content in this program. Determining whether this training could support other types of health workers is an important area for future investigation. Lastly, this study is largely guided by the content of the evidence-based COPSI program and accompanying manual and instructional content [14], as well as supplemented with additional key resources focused on effective counseling skills and empowering individuals living with disabilities [42,43,44]. While content experts have commented on the program design, there may be other important design considerations or essential features that may be omitted from the program developed in this study.

## 4. Conclusions

This preliminary study will break new ground by using widely available smartphone technology to support the acquisition of knowledge and clinical skills and developing capacity of community health workers for detecting and referring patients with schizophrenia in primary care settings in rural India. We hypothesize that this initial study will demonstrate success in addressing misconceptions about schizophrenia and empowering frontline community health workers with essential skills for identifying, referring, and responding to the needs of their patients with schizophrenia and their family members. Importantly, we anticipate that with the findings from this study, we will be ideally positioned to guide future efforts aimed at assessing how highly scalable digital training for community health workers can potentially address the multiple factors associated with greater disease burden due to schizophrenia in LMICs, including addressing misconceptions about the illness and supporting family caregivers [60], and ultimately reducing delays in the detection and referral for initiation of treatment for persons living with schizophrenia. These findings hold potential to inform the development of community-based services aimed at supporting the treatment and rehabilitation of persons living with schizophrenia and supporting their caregivers in LMICs.

## Figures and Tables

**Table 1:** Overview of the core program content in SARATHA.

Module No.	Module	Learning Objectives	Learning Outcomes
1	Introduction to Schizophrenia	To learn about schizophrenia and its symptoms To dispel misconceptions associated with schizophrenia To know the issue of stigma and discrimination To know the potential risk with schizophrenia cases To know the available treatments of schizophrenia To know the physical comorbidities in schizophrenia To know the phases of illness	Understand schizophrenia and its symptoms Have a clear understanding of facts, stigma and discrimination related to schizophrenia Clarity on available treatments of schizophrenia Able to recognise different phases of the illness
2	Disability and Rehabilitation	To understand rehabilitation and its need To know about disability To know about various disability schemes and benefits	Understand the importance of rehabilitation to improve the quality of life of the individual living with schizophrenia Understand the concept of disability and various schemes by the government. Skilled in the process of obtaining Disability Certificates and able to support patients and their families in obtaining the disability card
3	Understanding of COVID-19	To develop a basic understanding of COVID-19 To learn about vaccines To know how COVID-19 may impact persons living with schizophrenia and their Family	Understanding COVID-19 and its impact on persons living with schizophrenia and their families and regarding vaccination for COVID-19
4	Identifying Schizophrenia in the community	To know the schizophrenia symptom profile checklist/screening tool To learn how to identify schizophrenia in community	Skilled in recognizing schizophrenia cases in community Skilled in detecting patients with the help of screening tool
5	Mental Health First Aid (MHFA)	How to respond to and involve a family member or caregivers Managing suicide risk in schizophrenia To learn about Mental Health First Aid (MHFA) and how to deliver MHFA	Skilled in communicating with family members and providing mental health first aid. Able to assess suicide risk and its management
6	Making a referral	Understand the referral process How and whom to refer How to coordinate with clinicians and caregivers or family member How to do documentation and reporting Post referral follow-up	Skilled in referring cases from the community and communicating with clinicians and caregivers Able to educate family members/caregiver about the importance of referral Able to maintain a record of community processes and reporting

Note: The core program content in SARATHA was adapted primarily from the COPSI manual [14,41], which has been simplified into a 6-module program. Additional content for the program was adapted from the Department of Empowerment of Persons with Disability [42], The PREMIUM Counselling Relationship Manual [43], as well as the “Nuts and Bolts of Starting and Running Psychiatric Rehabilitation Services” manual developed by National Institute of Mental Health and Neurosciences [44].

## Data Availability

Data from this study will be made available upon request.
